# Optimisation of Recombinant Myrosinase Production in *Pichia pastoris*

**DOI:** 10.3390/ijms22073677

**Published:** 2021-04-01

**Authors:** Zuzana Rosenbergová, Kristína Kántorová, Martin Šimkovič, Albert Breier, Martin Rebroš

**Affiliations:** 1 Institute of Biotechnology, Faculty of Chemical and Food Technology, Slovak University of Technology, Radlinského 9, 812 37 Bratislava, Slovakia; zuzana.rosenbergova@stuba.sk (Z.R.); kristina.kantorova@stuba.sk (K.K.); 2 Institute of Biochemistry and Microbiology, Faculty of Chemical and Food Technology, Slovak University of Technology, Radlinského 9, 812 37 Bratislava, Slovakia; martin.simkovic@stuba.sk (M.Š.); albert.breier@stuba.sk (A.B.)

**Keywords:** myrosinase, *Pichia pastoris*, fermentation, upscale, *Arabidopsis thaliana*

## Abstract

Myrosinase is a plant defence enzyme catalysing the hydrolysis of glucosinolates, a group of plant secondary metabolites, to a range of volatile compounds. One of the products, isothiocyanates, proved to have neuroprotective and chemo-preventive properties, making myrosinase a pharmaceutically interesting enzyme. In this work, extracellular expression of TGG1 myrosinase from *Arabidopsis thaliana* in the *Pichia pastoris* KM71H (Mut^S^) strain was upscaled to a 3 L laboratory fermenter for the first time. Fermentation conditions (temperature and pH) were optimised, which resulted in a threefold increase in myrosinase productivity compared to unoptimised fermentation conditions. Dry cell weight increased 1.5-fold, reaching 100.5 g/L without additional glycerol feeding. Overall, a specific productivity of 4.1 U/L_medium_/h was achieved, which was 102.5-fold higher compared to flask cultivations.

## 1. Introduction

**Figure 1:**
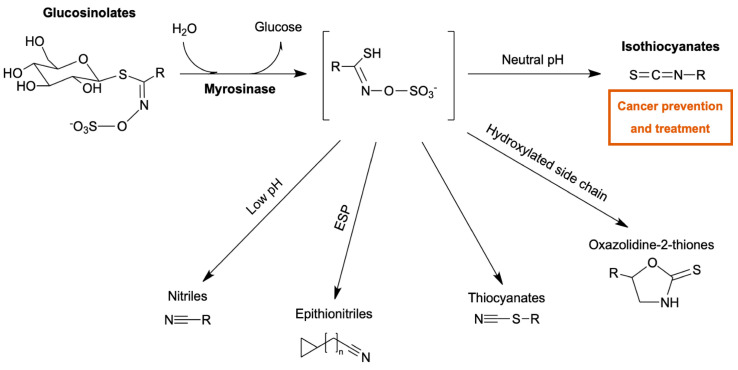
Scheme of glucosinolate hydrolysis. Bioactive products formation after myrosinase-catalysed hydrolysis of glucosinolates, which is affected by pH, the epithiospecifier protein (ESP), and the structure of the amino acid-derived side chain (R).

Myrosinase (EC 3.2.1.147), a β-thioglucosidase, is a part of the plant defence system. Depending on the reaction conditions, it catalyses the hydrolysis of glucosinolates into a range of products (Figure 1) [1]. One family of products, isothiocyanates (ITCs), is reported to have antimutagenic, neuroprotective, and chemo-preventive effects. ITCs have been previously linked with the reduction of cancer occurrence [2]. In addition to their cancer-preventive role, there is evidence showing their anti-tumorigenic action against different types of cancer, both in vitro and in vivo. Tumour inhibition by ITCs is mediated through the modulation of critical cancer-related pathways. Due to their low toxicity profile, ITCs are considered to be excellent chemotherapeutic candidates against tumour initiation and progression [3]. Moreover, increasing attention has been given to diet-derived molecules that could prevent or suppress carcinogenesis [4], which makes the glucosinolate-myrosinase system an interesting alternative to current cancer treatments.

Since its discovery more than 150 years ago, myrosinase has been identified in more than 15 different plants, gut microbes, and fungi. In plants, myrosinases are generally encoded by a multigene family [5]. *Arabidopsis thaliana* possesses six different myrosinase genes, while *Brassica napus* was reported to have at least 20 myrosinase isoenzymes, which are divided into three subclasses based on enzyme activity, solubility, and molecular weight [6,7]. Therefore, purification of these isoenzymes from their native host is very challenging. Recombinant production of plant myrosinases has proven to be a powerful tool in the production of a single myrosinase isoenzyme to facilitate their purification and characterisation. Moreover, for pharmaceutical applications, large quantities of myrosinase have to be produced, which would be impossible to achieve by isolating it from plants.

The first recombinant production of myrosinase was published by Chen and Halkier [7], who reported heterologous expression of active MYR1 myrosinase from *Brassica napus* in *Saccharomyces cerevisiae*. It was the first naturally complex-bound myrosinase to be produced with no cross-contamination by lectins [7]. This work was followed by Härtel and Brandt [8], who expressed MYR1 from *B. napus* in *Pichia pastoris*. The previously observed problems with myrosinase expression in *S. cerevisiae* were solved, including the toxicity of intracellular expression to *S. cerevisiae* cells and low expression levels.

*P. pastoris* has been used as an expression host for recombinant protein production for more than 20 years [9]. The gene of interest is usually placed under the control of the methanol-inducible AOX1 promoter, although many other promoters are available. As an eukaryotic organism, *P. pastoris* can perform posttranslational modifications without hyper-glycosylation (common for *S. cerevisiae*) and can secrete expressed proteins into cultivation medium. The ability to grow *P. pastoris* to high-cell densities on minimal media often leads to high yields of the desired protein [10].

As mentioned previously, myrosinases have been expressed in *P. pastoris* (Table 1), only in Mut^+^ strains and mostly intracellularly. For industrial applications, extracellular protein expression is desirable because it greatly simplifies downstream processing [11]. Only two cases of extracellular myrosinase production were published: TGG1 and TGG2 myrosinases from *A. thaliana* [12] and MYR1 myrosinase from *B. napus* [8]. In both cases, the production of myrosinase itself was not studied, optimised, or upscaled, and was mostly used for enzyme property studies.

In this paper, extracellular and high-cell density production of active TGG1 myrosinase from *A. thaliana* was studied in the *P. pastoris* Mut^S^ strain. Cultivation conditions were optimised, and the production was upscaled for the first time. Furthermore, an ascorbic acid-independent and kit-free method for effective transformant screening with glucose dehydrogenase was developed.

## 2. Results and Discussion

### 2.1. Recombinant Strain Selection and Myrosinase Activity Assay

The pPICZαA vector carrying the TGG1 gene encoding myrosinase from *A. thaliana* was successfully transformed into the *P. pastoris* KM71H strain. Transformed cells were plated on YPD plates with different Zeocin^TM^ concentrations. The clones with the highest Zeocin^TM^ concentration tolerance were subjected to 96 deep-well plate expression trials.

**Figure 2:**
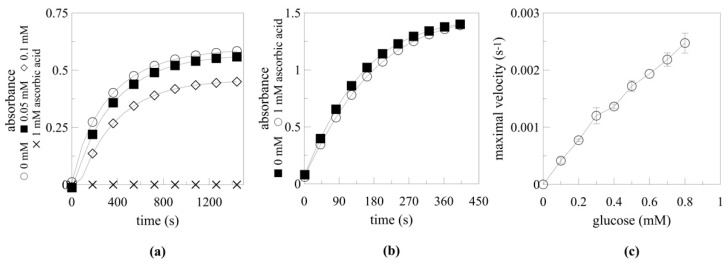
The effect of ascorbic acid on glucose determination assays. Inhibition of the colour development of glucose oxidase (GOD) kit by ascorbic acid (**a**). Ascorbic acid-independent glucose dehydrogenase (GDH) activity (**b**) and the dependence of maximal velocity in GDH catalysed reactions with various glucose concentrations (**c**).

The simple, ideally spectrophotometric method for clone screening is crucial for performing a microplate assay. Most methods for myrosinase activity measurement are based on the determination of released glucose (Figure 1), for which mainly glucose oxidase (GOD) kit is used. However, the main drawback of using GOD kit is the inhibition of colour release by ascorbic acid (Figure 2a), a known myrosinase cofactor [17,18]. An alternative to the GOD method is a two-step method based on the phosphorylation of glucose by hexokinase and its subsequent oxidation by glucose-6-phosphate dehydrogenase. The reduction of the NAD^+^ cofactor is then used for glucose determination. This method was proved to be ascorbic acid independent [17]; however, it usually requires a commercial kit with limited storage stability. Therefore, a single-step method using glucose dehydrogenase (GDH) was developed in this study. GDH was produced recombinantly in *Escherichia coli* according to Petrovičová et al. [19] and was stored for 10 months without major loss of activity. Similar to the previously mentioned method, the spectrophotometrically measured reduction of the NAD^+^ cofactor was used for GDH activity determination.

Using GDH for a microplate assay when screening transformants for myrosinase activity proved to be eligible since there were minimal effects of ascorbic acid (1 mM; Figure 2b) on GDH activity. Furthermore, less sinigrin needed to be applied since lower sample dilution was required for glucose determination to minimise the inhibiting effect of ascorbic acid. The released glucose concentration was determined based on a calibration curve (Figure 2c). When 0.115 mg/mL GDH with a specific activity of 14.6 U/mg was used, the calibration curve was linear up to 0.8 mM of glucose in the reaction mixture. The best clones were determined based on the highest GDH activity, reflecting the highest glucose concentration in the reaction mixture after 20 h.

Five clones with the highest myrosinase activity were selected for flask cultivation. The highest expressing clone was selected according to the highest myrosinase activity (0.0038 U/mL) with a dry cell weight (DCW) of 11.64 g/L and specific productivity of 0.040 U/L_medium_/h in the supernatant after 72-h of methanol induction.

### 2.2. High-Cell Density Cultivation and the Effect of Temperature on Myrosinase Expression

Flask cultivations of the recombinant *P. pastoris* Mut^+^ strain expressing TGG1 myrosinase from *A. thaliana* has been previously reported (Table 1). Due to the low biomass concentration, only low amounts of recombinant proteins are produced in flask cultivations of *P. pastoris* [20]. To improve this bottleneck, the process needs to be optimised in a fermenter with precise parameter regulation [21]. To our knowledge, myrosinase expression in a fermenter has never been reported. Therefore, high-cell density process in 350 mL mini fermenters (DASbox^®^ Mini Reactor Systems, Eppendorf, Germany) with a 150 mL working volume was optimised. In the glycerol-growth phase (approximately 20 h), the temperature was set to 30 °C and maintained for 24 h during the methanol adaptation phase. Then, continuous methanol feeding was started and the temperature was either kept at 30 °C or changed to 25 or 20 °C. Methanol additions were regulated automatically by the level of dissolved oxygen according to Markošová et al. [21].

**Figure 3:**
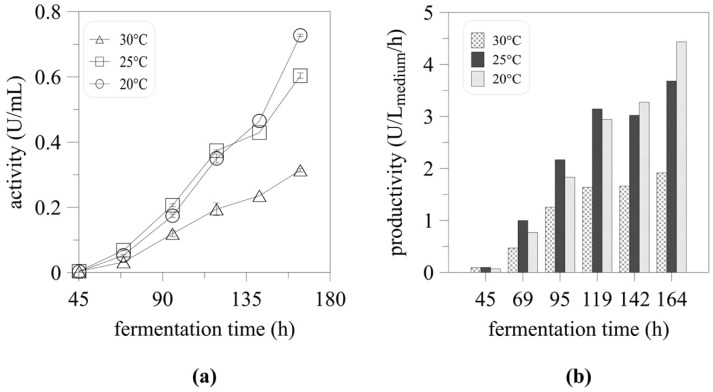
The effect of decreased induction temperature on myrosinase production in mini fermenters. Increase in myrosinase activity (**a**) and specific productivity (**b**) at decreased induction temperature in high cell density cultivation of *Pichia pastoris* KM71H (Mut^S^) in mini fermenters.

Lowering the cultivation temperature from 30 to 25 or 20 °C during myrosinase expression resulted in a significant increase in myrosinase activity (Figure 3a) and specific productivity (Figure 3b) which increased 1.9- and 2.3-fold. The maximum biomass concentration also increased from 66.2 to 109.9 and 98.2 g/L DCW, respectively. The positive effect of lower expression temperatures has already been reported for other proteins produced in *P. pastoris*. The specific productivity of the 3H6 Fab fragment increased threefold when expressed at 20 °C by the recombinant *P. pastoris* Mut^+^ strain [22]. A similar effect of decreased cultivation temperature was reported for human interleukin-10, laccase, and polygalacturonate lyase [23,24,25]. An improvement in scFv single-chain antibody expression was also achieved by lowering the cultivation temperature to 15 °C [26].

The increase in myrosinase activity and biomass yield possibly resulted from reduced cell stress. Stress can be caused by the retainment of improperly folded proteins in the endoplasmic reticulum or depletion of precursors and energy due to the increased transcript levels at higher cultivation temperatures. Reduced cell stress reflects decreased cell lysis and possibly lesser amounts of intracellular proteases released to the cultivation medium. This can significantly improve recombinant protein yield if the product is susceptible to proteolytic decay [23,27].

### 2.3. Cultivation Upscale

For the industrial application of any enzyme, upscaling its production to larger volumes is always desirable. Hence, the high-cell density cultivation was upscaled to a 3 L fermenter with a 1.5 L working volume, and the effect of decreased temperature on myrosinase activity was verified (30 °C and 20 °C induction).

**Figure 4:**
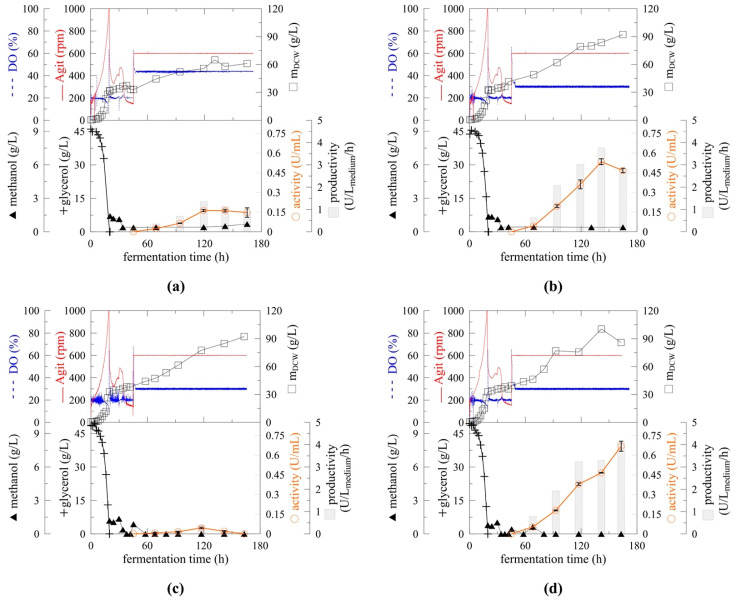
Temperature and pH optimisation of upscaled high-cell density cultivation of *Pichia pastoris* expressing myrosinase. Fermentation of *P. pastoris* KM71H (Mut^S^) expressing *At*TGG1 myrosinase at 30 (**a**) and 20 °C (**b**) at pH 5; and at pH 4 (**c**), and 6 (**d**) at 20 °C.

Results obtained from upscaled fermentation correlated well with the results from mini fermenters described above. The positive effect of decreased temperature resulted in a 1.5-fold increase in biomass concentration and a 2.8-fold increase in specific productivity. However, the highest myrosinase activity and specific productivity were achieved at different times of fermentation. When the induction temperature was set to 30 °C (Figure 4a), a maximal productivity of 1.36 U/L_medium_/h was achieved after 120 h and slightly decreased with prolonged fermentation. When the induction temperature was set to 20 °C (Figure 4b), maximal productivity reached 3.76 U/L_medium_/h after 142 h of fermentation, which was 2.8-fold higher than the highest productivity achieved at 30 °C. Over the next 24 h, myrosinase activity and specific productivity decreased by 25%. This phenomenon was observed repeatedly when expressing myrosinase using this fermentation setup (data not shown).

The decrease in myrosinase activity after 140 h could have been a result of proteolytic degradation since a decrease in total protein concentration in the culture supernatant was also observed (from 333.7 to 256.1 mg/L). Protease activity toward azoalbumin has been previously determined in supernatants of the *P. pastoris* Mut^S^ strain. Proteolytic activities indicated three types of proteases present in methanol-induced supernatants: aspartic (pH 3.5–4.5), cysteine (pH 5.5–7.5), and serine (pH 10) [26]. This might also explain the drop in myrosinase activity at the end of the fermentation process.

### 2.4. Effect of pH on Myrosinase Expression

Altering the cultivation conditions is commonly done when the target protein is a subject of proteolytic degradation. Lowering the cultivation temperature, adding amino acid-rich supplements and protease inhibitors, or using protease deficient strains has proven to be efficient in many cases [28]. Each recombinant protein can be sensitive to different proteases in the culture supernatant, depending on its amino acid sequence. Therefore, the optimisation of cultivation conditions is required for each protein individually. Since proteases found in methanol-induced *P. pastoris* supernatants have different pH optima [26], altering the pH of the cultivation medium can significantly improve recombinant protein yield through inactivation of the target protease. Although the long-term cultivation of yeast at non-optimal conditions can increase environmental stress, *P. pastoris* is known to tolerate a broad pH range (3.0 to 7.0) without significant effects on its growth [27,28].

High-cell density fermentation with the same fermentation setup as described above was performed to determine the effect of pH on myrosinase expression and activity. Similar to temperature optimisation, standard conditions (30 °C, pH 5) were maintained during the glycerol-growth phase and methanol adaptation. However, in the methanol induction phase, the cultivation temperature was set to 20 °C and pH either spontaneously decreased and was maintained at 4 (Figure 4c), or was adjusted to 6 (Figure 4d).

As expected, altering the cultivation pH during myrosinase expression had no significant effect on biomass growth. Biomass yield after 164 h was 92.3 (pH 4) and 85.6 g/L DCW (pH 6). However, significant differences in myrosinase activity at different induction pHs were observed. A lower pH of 3–5 for secreted protein production in *P. pastoris*, as recommended by the host provider, resulted in very low activity of the produced myrosinase (Figure 4c). The maximal activity of 0.045 U/mL was reached after 120 h of cultivation, representing only 7% of the maximal activity achieved in the fermentation at pH 6 (Figure 4d). Based on Shi et al. [26], aspartic proteases are active at pH 4. To eliminate the possibility of myrosinase being susceptible to this group of proteases, fermentation at pH 3 was performed. However, these conditions lead to a complete loss of myrosinase activity (data not shown). These results indicate that the decrease in myrosinase activity is a protein stability problem rather than proteolytic degradation. An increase in the *P. pastoris* protein expression pH proved to be beneficial also for other proteins: scFv single-chain antibody (pH 7.5–8), mouse epidermal factor (pH 6) and Fc fusion protein (pH 7.2) [26,29,30]. An increase in pH to 6 also solved the problem observed previously when myrosinase activity decreased after 140 h of fermentation (Figure 4b). Activity increased linearly until 163 h when fermentation was terminated (Figure 4d). A maximal productivity of 4.1 U/L_medium_/h was achieved, which was threefold higher than the maximal productivity reached with unoptimised fermentation conditions (30 °C, pH 5). When compared with flask cultivation, more than a 100-fold increase in productivity was achieved. The parameters of myrosinase expression in *P. pastoris* is summarised in Table 2.

### 2.5. Enzyme Parameters

#### 2.5.1. Temperature and pH Optimum

Temperature and pH profiles were previously determined for *At*TGG1, *At*TGG4, and *At*TGG5 myrosinase produced in *P. pastoris* [13]. However, characterisation was performed with intracellularly produced, IMAC (Immobilised Metal Affinity Chromatography) purified enzymes. pH and temperature optima in this work were determined for an impurified enzyme using a desalted culture supernatant.

**Figure 5:**
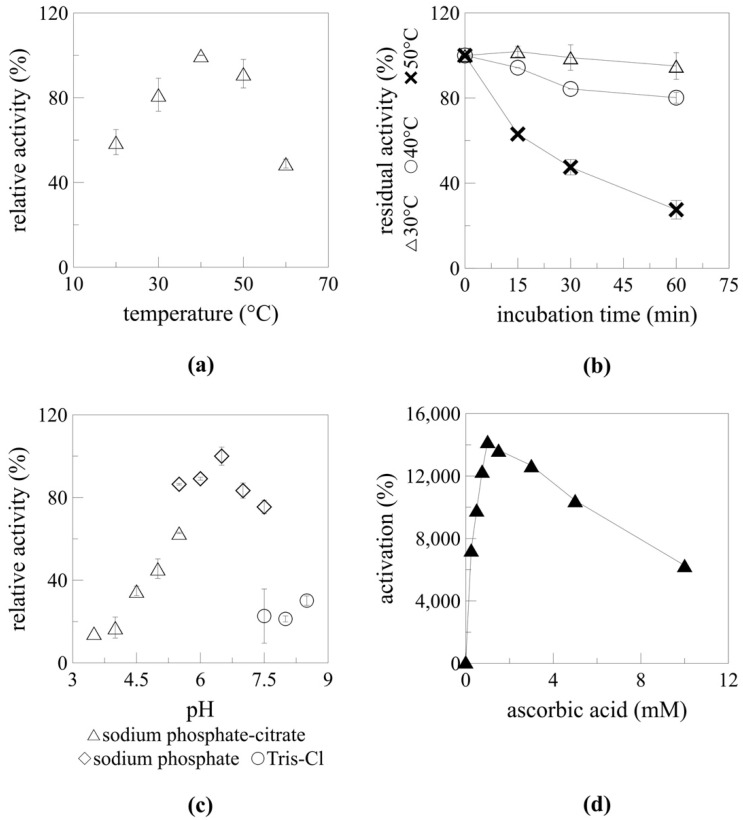
Catalytic properties of recombinant myrosinase. Temperature profile (**a**), operational temperature stability (**b**), pH profile (**c**), and ascorbic acid-dependent activation (**d**) of recombinant *At*TGG1 myrosinase in the form of culture supernatant.

The highest myrosinase activity was detected at 40 °C (Figure 5a). Almost 91% of this activity was detected at 50 °C and approximately 81% at 30 °C. These results correlated well with previously published results [13]. However, almost 50% of the maximum myrosinase activity even at 60 °C was detected, while Andersson et al. [13] reported no measurable activity at this temperature. The operational stability of myrosinase at 30, 40, and 50 °C during 1-h incubation was also determined (Figure 5b). As expected, the lowest decrease in myrosinase activity was observed at 30 °C, when 95% of the initial activity was retained even after a 1-h incubation. A 20% decrease and 73% drop in the initial activity was detected after 1-h incubation at 40 and 50 °C, respectively. These results confirm that *At*TGG1 myrosinase is less stable at higher temperatures than other myrosinase enzymes from *A. thaliana*; *At*TGG4 and *At*TGG5 myrosinase have temperature optima at 60 and 70 °C, respectively [13].

It has been previously reported that myrosinases exhibit activity in a broad pH range [8,13,16,31]. Three buffers with a buffering capacity at different pH ranges were selected to determine the pH profile: sodium citrate-phosphate (3.5–5.5), sodium phosphate (5.5–7.5), and Tris-HCl (7.5–8.5). The pH optimum for *At*TGG1 myrosinase was 6.5 in sodium phosphate buffer (Figure 5c). A similar pH optimum was determined for native *At*TGG1 myrosinase [31]. Only limited activity was measured below pH 4 (less than 17%) and above pH 7.5 (approximately 22% in Tris-HCl buffer).

A higher myrosinase activity was detected in sodium phosphate buffer than in Tris-HCl buffer at pH 7.5. It has been previously reported that Tris inhibits the activity of α-galactosidases, trehalases, and disaccharidases [32,33,34,35]. The same phenomenon was observed for sodium phosphate-citrate buffer at pH 5.5, which can be explained by zinc chelation by citrate; this was previously reported for carboxypeptidase A [36].

#### 2.5.2. Myrosinase Activation by Ascorbic Acid

The mechanism of myrosinase activation by ascorbic acid was described by Burmeister et al. [18], who found that ascorbic acid substitutes the function of the catalytic base in the myrosinase active site. Myrosinase activity was measured with ascorbic acid ranging from 0 to 10 mM in the reaction mixture (Figure 5d). The highest activation was observed at 1 mM of ascorbate when a 142-fold increase in myrosinase activity was detected. Further increases in ascorbate concentration resulted in a lower activation of myrosinase, which corresponded with previous observations [13,15].

Wang et al. [15] and Andersson et al. [13] reported complete inactivation of TGG2 myrosinase from *Carica papaya* and *At*TGG1 myrosinase by ascorbic acid at 4 and 5 mM, respectively. However, in this study myrosinase was activated at 5 mM ascorbate (104-fold), as well as at 10 mM (63-fold). As mentioned previously, 0.05 mM ascorbic acid is known to interfere with GOD for glucose determination [17], which is usually used for myrosinase activity determination. This has also been shown by Nong et al. [16], who used two methods for glucose determination (GOD and hexokinase kit) when defining the ascorbic acid dependence of TGG1 myrosinase from *Carica papaya*. The results were comparable to 1 mM ascorbic acid in the reaction mixture. Further increases in the ascorbic acid concentration led to different results for each method [16]. To avoid this underestimation, a more precise determination of myrosinase activity by sinigrin biotransformation and its HPLC evaluation was applied in this study.

### 2.6. Storage of Myrosinase

One of the key aspects of enzyme application is its long-term stability. The long-term stability of the impurified, cell-free myrosinase supernatant was tested. The fermentation supernatant was microfiltered, concentrated, and desalted on a membrane (30 kDa cut-off) before storage. The effect of glycerol addition on myrosinase stability was also tested.

**Figure 6:**
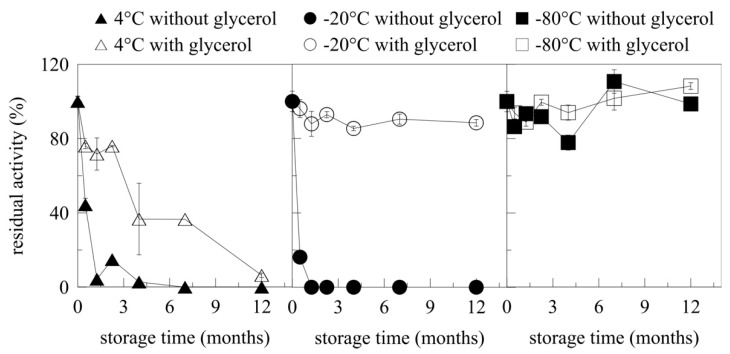
Long-term storage of recombinant myrosinase. Residual activity of recombinant *At*TGG1 myrosinase stored in the form of culture supernatant with and without glycerol at 4, −20 and −80 °C.

The storage of myrosinase at 4 and −20 °C with no glycerol proved to have a detrimental effect on its activity (Figure 6). After five weeks, myrosinase stored at 4 °C was completely inactivated. Freezing the supernatant at −20 °C lead to complete inactivation of myrosinase after only two weeks of storage. It is known that freezing the sodium phosphate buffer results in uneven ice formation, which causes a drop in pH (more than three units) and increases the ionic strength [37,38]. This is possibly the reason for rapid inactivation of myrosinase stored at −20 °C.

As expected, the addition of glycerol [50% (*v/v*) final concentration] significantly enhanced the long-term stability of recombinant myrosinase. After 12 months of storage, the supernatant stored at −20 °C in 50% (*v/v*) glycerol had 90% of its original activity. No significant changes in myrosinase activity were observed when storing the supernatant at −80 °C. After 12 months, myrosinase exhibited 99% residual activity without glycerol and 108% residual activity with added glycerol. Hence, myrosinase can be stored at −80 °C without loss of its activity even without the need for use of any cryo-preservative agent. Arsiccio et al. [37] described a model for the stability of different proteins during freezing. A high cooling rate, when smaller ice crystals are formed, should be applied if the protein is poorly stable in the solution. This is probably true for myrosinase based on the results obtained at 4 and −20 °C [37].

Markošová et al. [21] reported the storage of α-L-rhamnosidase produced recombinantly in *P. pastoris*. The enzyme stored at 4 °C in the form of a cell-free fermentation supernatant was stable for 18 months, since 73% of its original activity was retained. These results differed from those obtained in this paper, since myrosinase stored at 4 °C was completely inactivated after five weeks of storage. This demonstrates that, similar to the optimisation of fermentation parameters, storage needs to be optimised individually for each enzyme produced in *P. pastoris*.

## 3. Materials and Methods

### 3.1. Microorganism and Media

*P. pastoris* KM71H (Mut^S^) and *E. coli* DH5α were used in this work. The strains were cryopreserved as follows: *P. pastoris* in YPD [Yeast extract/Peptone/Dextrose medium; 1% (*w/v*) yeast extract, 2% (*w/v*) peptone, and 2% (*w/v*) glucose] in 30% (*v/v*) glycerol and *E. coli* in LB [Lysogeny Broth; 1% (*w/v*) NaCl, 1% (*w/v*) tryptone, and 0.5% (*w/v*) yeast extract] in 50% (*v/v*) glycerol. Microorganisms were also cultivated on plates containing 2% (*w/v*) agar and 50 mg/L Zeocin^TM^ (InvivoGen, San Diego, CA, USA).

Cultivations in 96-deep well plates were performed in BMD [Buffered Minimal Dextrose medium; 1.34% (*w/v*) YNB (Yeast Nitrogen Base), 4 × 10^−5^% (*w/v*) biotin, 1% (*w/v*) glucose, and 200 mM potassium phosphate buffer, (pH 6)], BMM2 (Buffered Minimal Methanol medium; 1.34% (*w/v*) YNB, 4.10^−5^% (*w/v*) biotin, 1% (*v/v*) methanol, 200 mM potassium phosphate buffer (pH 6)), and BMM10 (Buffered Minimal Methanol medium; 1.34% (*w/v*) YNB, 4.10^−5^% (*w/v*) biotin, 5% (*v/v*) methanol, and 200 mM potassium phosphate buffer (pH 6)) media according to Weis et al. [39].

For flask experiments, BMGY (Buffered Glycerol-complex Medium; 1% (*w/v*) yeast extract, 2% (*w/v*) peptone, 1.34% (*w/v*) YNB, 4 × 10^−5^% (*w/v*) biotin, 1% (*v/v*) glycerol, and 100 mM potassium phosphate (pH 6)) and BMMH (Buffered Minimal Methanol Medium; 1.34% (*w/v*) YNB, 4 × 10^−5^% (*w/v*) biotin, 0.5% (*v/v*) methanol, and 100 mM potassium phosphate (pH 6)) media were used.

Fermentation experiments were performed in BSM medium (Basal Salt Medium; per 1 L: 0.93 g CaSO_4_, 18.2 g K_2_SO_4_, 14.9 g MgSO_4_. 7 H_2_O, 4.13 g KOH, 40 g glycerol, and 26.7 mL 85% H_3_PO_4_) supplemented with 4.35 mL PTM1 (trace salts solution; per 1 L: 6 g CuSO_4_ 0.5 H_2_O, 0.08 g NaI, 3 g MnSO_4_.H_2_O, 0.2 g Na_2_MoO_4_.2 H_2_O, 0.02 g H_3_BO_3_, 0.5 g CoCl_2_, 20 g ZnCl_2_, 65 g FeSO_4_.7H_2_O, 0.2 g biotin, and 9.2 g H_2_SO_4_) per litre of BSM medium. PTM_1_ solution was also added to the methanol feed in high-cell density fermentations (12 mL PTM_1_ per litre of methanol).

### 3.2. Recombinant Strain Preparation

Plasmid pPICZαA containing the codon-optimised TGG1 gene for myrosinase from *A. thaliana* (GenBank Accession no. CAA55786.1) was purchased from Generay Biotech Co., Ltd. (Shanghai, China). Plasmid pPICZαA-*myr* was transformed to *E. coli* DH5α cells. Plasmid DNA was extracted using the Genopure Plasmid Midi Kit (Roche Molecular Systems, Inc., Pleasanton, CA, USA) and linearised with *Sac*I (Fast Digest, ThermoFisher Scientific, Waltham, MA, USA). Approximately 5 μg of linearised plasmid was transformed to *P. pastoris* KM71H competent cells by electroporation. Competent *P. pastoris* cells were prepared by a condensed protocol according to [40]. Transformed cells were plated on YPD with various Zeocin^TM^ concentrations (100–250 mg/L) and cultivated at 30 °C for 48 h.

### 3.3. Cultivation in Deep Well Plates

*P. pastoris* transformants were screened for myrosinase activity in a 96-deep well plate according to the protocol used by Markošová et al. [41]. Each well, containing 250 μL of BMD medium, was inoculated by a single colony and cultivated at 30 °C and 300 rpm. After 48 h, colonies were plated on the YPD plate and methanol induction was initiated by adding 250 μL of BMM2 medium to each well. In 12-h intervals, 50 μL of BMM10 medium was added to each well. After approximately 92 h, the deep well plate was centrifuged (1900× *g*, 10 °C, 10 min), and the supernatants were screened for myrosinase activity.

### 3.4. Microplate Assay

The myrosinase activity assay for best transformant determination was performed in a 96-well microplate (Sarstedt, Germany) using a Varioskan^®^ Flash microplate reader (ThermoFisher Scientific, Waltham, MA, USA). The reaction mixture (100 μL), containing 50 μL of cell-free supernatant, 0.3 mM sinigrin (Sigma-Aldrich, Saint-Louis, MO, USA), 0.5 mM ascorbic acid, and 0.1 M sodium phosphate buffer (pH 6.5), was incubated in a microplate reader at 30 °C and shaken at 240 spm (8 mm diameter). After 20 h, the released glucose was determined by adding 50 μL of solution containing 1.5 mM NAD^+^, 0.347 mg/mL glucose dehydrogenase (GDH, 14.6 U/mg with NAD^+^), and 0.1 M potassium phosphate buffer (pH 7). The reaction was performed at 30 °C, shaken at 240 spm (8 mm diameter), and measured at 340 nm for 20 min. Maximal velocity was calculated by Varioskan^®^ Flash software (ThermoFisher Scientific, Waltham, MA, USA).

### 3.5. Flask Cultivation

Flask cultivation was performed in 500 mL shake-flasks. First, 100 mL of BMGY medium was inoculated with a single *P. pastoris* colony and cultivated at 30 °C and 200 rpm for 18 h. Then, the cells were harvested by centrifugation (13,751× *g*, 10 °C, 10 min) and resuspended in 100 mL of BMMH medium. Pure methanol (100 μL) was added to BMMH medium 3 times a day. Cultivation was terminated after 90 h (a 72-h induction with methanol). Supernatants from the cultivation of the untransformed *P. pastoris* KM71H strain and a *P. pastoris* KM71H strain expressing a different gene were used as controls.

### 3.6. High-Cell Density Cultivation and Scale-Up

Fermentations were performed in 350 mL mini fermenters (DASbox^®^ Mini Reactor Systems, Eppendorf, Germany). The cultivation procedure was described previously [21]. Briefly, an overnight preculture (BMGY medium, 30 °C, 200 rpm, 17 h) was used to inoculate (5% (*v/v*) inoculum, OD_600_: 14–16) 150 mL BSM medium supplemented with 0.653 mL PTM_1_. A 20% dissolved oxygen (DO) saturation was maintained by an agitation cascade (50–2000 rpm). After glycerol depletion (approx. 22 h), two methanol pulses to 3 g/L were performed, and after the second methanol depletion (approximately 45 h), continuous methanol feeding was started. Agitation was fixed at 1000 rpm, the DO control was turned off, and a third methanol pulse was performed. Methanol feeding was controlled by an automated program that switched the pump on when DO saturation was between 30–40%, maintaining the level of methanol below 3 g/L. Agitation parameters were altered from the original protocol [21] due to different vessel construction. The fermentation upscale was performed according to the original protocol in a 3 L fermenter (New Brunswick^TM^ BioFlo^®^ 115, Eppendorf, Germany) with 1.5 L working volume. Temperature and pH were set to different values based on each experiment.

In all fermentation experiments, antifoam (Struktol J650, Schill + Seilacher, Germany) was added directly to the BSM medium before sterilisation (200 μL of antifoam per 1.5 L of BSM medium). No additional antifoam was needed throughout the fermentation.

After fermentation, the cultivation medium was centrifuged (13,751× *g*, 10 °C, 15 min), the biomass was discarded, and the supernatant was either filtered through 0.45 μm mixed cellulose ester membrane filter (ADVANTEC^®^ MFS, Inc., Japan) or subjected to tangential membrane filtration on a 0.44 μm membrane (TANGEN XTM PRO PDn Cassette, HyStream, REPLIGEN, Waltham, MA, USA) using ÄKTA flux (GE Healthcare, Chicago, IL, USA). The cell-free supernatant was concentrated and desalted with Amicon^®^ Ultra Centrifugal Filters with a 30 kDa cut-off (Merck Milipore, Burlington, MA, USA) or using a 30 kDa cut-off membrane (TANGEN XTM PRO PDn Cassette, ProStream, REPLIGEN, Waltham, MA, USA) with 50 mM sodium phosphate buffer (pH 6.5).

### 3.7. Myrosinase Activity Assay

The standard reaction mixture (500 μL) for the myrosinase activity assay contained 100 μL cell-free supernatant, 0.6 mM sinigrin, 1 mM ascorbic acid, and 50 mM sodium phosphate buffer (pH 6.5). The reaction was performed in a thermomixer (Thermomixer R, Eppendorf, Germany) at 30 °C and 600 rpm. Samples were taken at different times, and the reaction was terminated by boiling the reaction mixture for 5 min. The sinigrin concentration was then analysed by HPLC according to Tsao et al. [42]. One unit of myrosinase activity was defined as the amount of enzyme catalysing the conversion of 1 μmol sinigrin per minute in 50 mM sodium phosphate buffer at pH 6.5 and 30 °C.

Reaction conditions were changed for temperature and pH profile determination and ascorbic acid activation measurement. For pH profile determination, 50 mM sodium phosphate-citrate (pH 3.5–5.5), 50 mM sodium phosphate (pH 5.5–7.5), and 50 mM Tris-HCl (pH 7.5–8.8) were used. In temperature profile measurements, a temperature range of 20–60 °C was tested. Myrosinase activation was tested with 0, 0.25, 0.5, 0.75, 1, 1.5, 3, 5, and 10 mM ascorbic acid.

Operational stability was determined during one-hour incubation of myrosinase at 30 °C, 40 °C, and 50 °C. Samples were taken every 15 min. Then, a standard reaction was used for myrosinase activity determination.

### 3.8. Analysis

Biomass growth was controlled by optical density (OD_600_) measurement at 600 nm (BioSpectrophotometer, Eppendorf, Germany). A calibration curve was then used to calculate the dry cell weight (DCW): DCW (g/L) = 0.2001 × OD_600_ + 0.1075 [21].

The concentration of glycerol and methanol during fermentation was determined in the cell-free supernatant by HPLC (Agilent Technologies 1220 Infinity LC System with Agilent Technologies 1260 Infinity RI detector, Agilent Technologies, Santa Clara, CA, USA) using WATREX Polymer IEX H form 8 μm (250 × 8 mm column) and WATREX Polymer IEX H form 8 μm (40 × 8 mm guard column). A flow rate of 0.8 mL/min of 9 mM sulfuric acid at 45 °C was used.

The sinigrin concentration was determined by HPLC (Agilent 1260 Infinity LC System with Quaternary Pump and UV detector, Agilent Technologies, Santa Clara, CA, USA) according to Tsao et al. [42]. A Phenomenex Gemini^®^ NX 5μm C18 110Å (150 × 4.6 mm) was used at 30 °C. Sinigrin was purchased from Sigma-Aldrich (Saint-Louis, MO, USA).

The protein concentration in cell-free supernatants was determined by the Bradford method [43]. The Bradford reagent was purchased from Sigma-Aldrich (Saint-Louis, MO, USA).

## 4. Conclusions

High-cell density cultivation of the *P. pastoris* KM71H (Mut^S^) strain expressing TGG1 myrosinase from *A. thaliana* was performed for the first time. It is the first report of this type of strain used for myrosinase expression. The importance of producing myrosinase in fermenters was demonstrated since the productivity increased 102.5-fold compared to flask cultivation. Optimisation of cultivation temperature and pH led to a threefold increase in fermentation productivity. The produced myrosinase can be stored at −80 °C without a significant loss of activity for 12 months.

## Figures and Tables

**Table 1:** Recombinant expression of various myrosinase genes in *Pichia pastoris*.

Gene Donor	Gene	Expression Vector	Expression Host	Reference
*Arabidopsis thaliana*	TGG1, TGG2	pHIL-S1 (extracellular)	*P. pastoris* GS115 (Mut^+^)	[12]
*Arabidopsis thaliana*	TGG1, TGG2 TGG4, TGG5	pPIC3.5K (intracellular)	*P. pastoris* GS115 (Mut^+^)	[13]
*Armoracia rusticana*	ArMY2	pPIC3.5K (intracellular)	*P. pastoris* GS115 (Mut^+^)	[14]
*Brassica napus*	MYR1	pHIL-S1 (extracellular)	*P. pastoris* GS115 (Mut^+^)	[8]
*Carica papaya*	CpTGG2	pPIC3.5 (intracellular)	*P. pastoris* GS115 (Mut^+^)	[15]
*Carica papaya*	CpTGG1	pPIC3.5 (intracellular)	*P. pastoris* GS115 (Mut^+^)	[16]

**Table 2:** Summary of *Pichia pastoris* fermentations optimizing myrosinase expression.

	Conditions	Total Protein (mg/L)	Maximal DCW (g/L)	Maximal Activity (U/mL)	Activity after 163 h (U/mL)	Maximal Productivity (U/L_medium_/h)	Productivity after 163 h (U/L_medium_/h)
1	30 °C, pH 5	267.3	64.9	0.16	0.15	1.35	0.89
2	20 °C, pH 5	256.1	92.05	0.53	0.47	3.02	2.83
3	20 °C, pH 4	383.0	92.35	0.045	0	0.39	0
4	20 °C, pH 6	286.5	100.5	0.67	0.67	4.10	4.10

## Data Availability

The data presented in this study are available on request from the corresponding author.
